# High incidence of gallstones after Roux-en-Y reconstruction gastrectomy in gastric cancer: a multicenter, long-term cohort study

**DOI:** 10.1097/JS9.0000000000001136

**Published:** 2024-02-06

**Authors:** Baoping Zhang, Peng Nie, Yanyan Lin, Zelong Ma, Guogang Ma, Yongjin Wang, Yuhu Ma, Jinyu Zhao, Jinduo Zhang, Ping Yue, Ningzu Jiang, Xianzhuo Zhang, Liang Tian, Linzhi Lu, Jinqiu Yuan, Wenbo Meng

**Affiliations:** a The First Clinical Medical College, Lanzhou University; b Department of Anesthesiology; c Department of General Surgery, The First Hospital of Lanzhou University, Lanzhou; d Department of Gastric Surgery; e Department of Gastroenterology, Gansu Wuwei Tumor Hospital, Wuwei, Gansu; f Clinical Research Center, The Seventh Affiliated Hospital, Sun Yat-sen University, Shenzhen, Guangdong, People’s Republic of China

**Keywords:** gallstones, gastrectomy, gastric cancer, risk factors, Roux-en-Y reconstruction

## Abstract

**Background:**

Roux-en-Y reconstruction is a common anastomosis technique during gastrectomy in gastric cancer. There is a lack of studies on gallstones after Roux-en-Y reconstruction gastrectomy. This study investigated the incidence and potential risk factors associated with gallstones after Roux-en-Y reconstructive gastrectomy in gastric cancer.

**Methods:**

The study analyzed data from gastric cancer who underwent radical gastrectomy and Roux-en-Y reconstruction at two hospitals between January 2014 and December 2020. The patients fall into distal and total gastrectomy groups based on the extent of gastrectomy. The cumulative event probability curve was plotted using the Kaplan–Meier, and differences in gallstone between groups were evaluated using the Log-Rank. Propensity score matching was applied to construct a balanced total versus distal gastrectomies cohort. A Cox regression was employed to analyze the risk factors for gallstones after Roux-en-Y reconstructive gastrectomy in gastric cancer. Further subgroup analysis was performed.

**Results:**

Five hundred thirty-one patients were included in this study, 201 in the distal gastrectomy group and 330 in the total gastrectomy. During the follow-up, gallstones occurred in 170 cases after gastrectomy, of which 145 cases accounted for 85.29% of all stones in the first two years after surgery. Then, to reduce the impact of bias, a 1:1 propensity score matching analysis was performed on the two groups of patients. A total of 344 patients were evaluated, with each subgroup comprising 172 patients. In the matched population, the Cox regression analysis revealed that females, BMI ≥23 kg/m^2^, total gastrectomy, No.12 lymph node dissection, and adjuvant chemotherapy were risk factors for gallstones after Roux-en-Y reconstructive gastrectomy. Subgroup analysis showed that open surgery further increased the risk of gallstones after total gastrectomy.

**Conclusion:**

The incidence of gallstones increased significantly within 2years after Roux-en-Y reconstructive gastrectomy for gastric cancer. Patients with these risk factors should be followed closely after gastrectomy to avoid symptomatic gallstones.

## Introduction

Highlights

- The incidence of gallstones after Roux-en-Y reconstructive gastrectomy for gastric cancer is high and primarily within 2 years after gastrectomy.
- Gallstones are more likely to occur after total gastrectomy than distal gastrectomy, especially in gastric cancer patients undergoing open surgery.
- Female, BMI ≥23 kg/m^2^, total gastrectomy, lymph node dissection of the hepatoduodenal ligament (No.12), and adjuvant chemotherapy are risk factors for the development of gallstones after Roux-en-Y reconstructive gastrectomy.

Gastric cancer is the fifth most common cancer and the fourth leading cause of cancer-related deaths worldwide^1^. The primary treatment method for resectable gastric cancer is radical gastrectomy. This procedure can disrupt the normal physiological pathways, affecting gastrointestinal function and increasing the risk of complications. Among the long-term follow-ups after gastrectomy, gallstone is a common complication with a 7.4–40% prevalence^2–5^. The mechanism of gallstones after gastrectomy remains uncertain. It might be associated with the severed vagus nerve and anatomical changes, leading to impaired gallbladder contraction and changes in intestinal hormone^6,7^. These factors ultimately contribute to the alterations in bile composition, promoting gallstone formation postgastrectomy.

The survival time for gastric cancer after gastrectomy has increased due to early diagnosis, comprehensive perioperative treatment, and improved surgical technology^8,9^. However, gallstones after gastrectomy can potentially result in acute cholecystitis, which requires surgical intervention, otherwise it can affect the patient's quality of life. The anatomical changes and abdominal adhesions from gastrectomy heighten the likelihood of biliary tract injury in cholecystectomy, making cholecystectomy more difficult postgastrectomy. Consequently, it is crucial to investigate gallstone-related issues following gastrectomy to give valuable findings for prevention and therapy.

Current research on gallstones after gastrectomy is limited, especially in the presence of gallstones after a specific anastomotic technique in gastrectomy. Roux-en-Y reconstruction, a commonly employed anastomosis technique during gastrectomy, results in decreased secretion of gastric acid and pepsin, encourages gastric emptying, prevents alkaline reflux, and mitigates the risk of reflux gastritis^10^. We designed a retrospective cohort study to explore the incidence and risk factors related to gallstones after Roux-en-Y reconstruction gastrectomy in gastric cancer to complement the research gaps in the field.

## Material and method

### Study design and participants

This study is a two-center, retrospective cohort study, divided into a distal gastrectomy group and total gastrectomy according to the extent of gastrectomy to investigate the incidences and potential risk factors of gallstones after Roux-en-Y reconstruction gastrectomy in gastric cancer. This study was complied with Strengthening the Reporting of Cohort Studies in Surgery (STROCSS) guideline^11^ (Supplemental Digital Content 1, http://links.lww.com/JS9/B815).

This study used data from patients who underwent surgical intervention for gastric cancer from January 2014 to December 2020 in the Department of General Surgery of the First Hospital of Lanzhou University and the Department of Gastric Surgery of the Gansu Wuwei Tumor Hospital. The applied exclusion criteria were as follows: (1) Not Roux-en-Y reconstruction; (2) a history of upper abdominal surgeries, such as gastrectomy and cholecystectomy; (3) preoperative gallbladder diseases, including gallstones, gallbladder polyps, cholecystitis; (4) a previous history of malignant tumors; (5) emergency or palliative surgeries; (6) incomplete case data.

The study was approved by the ethics committee and registered at clinicaltrials.gov.

### Surgical procedures

**Figure 1:**
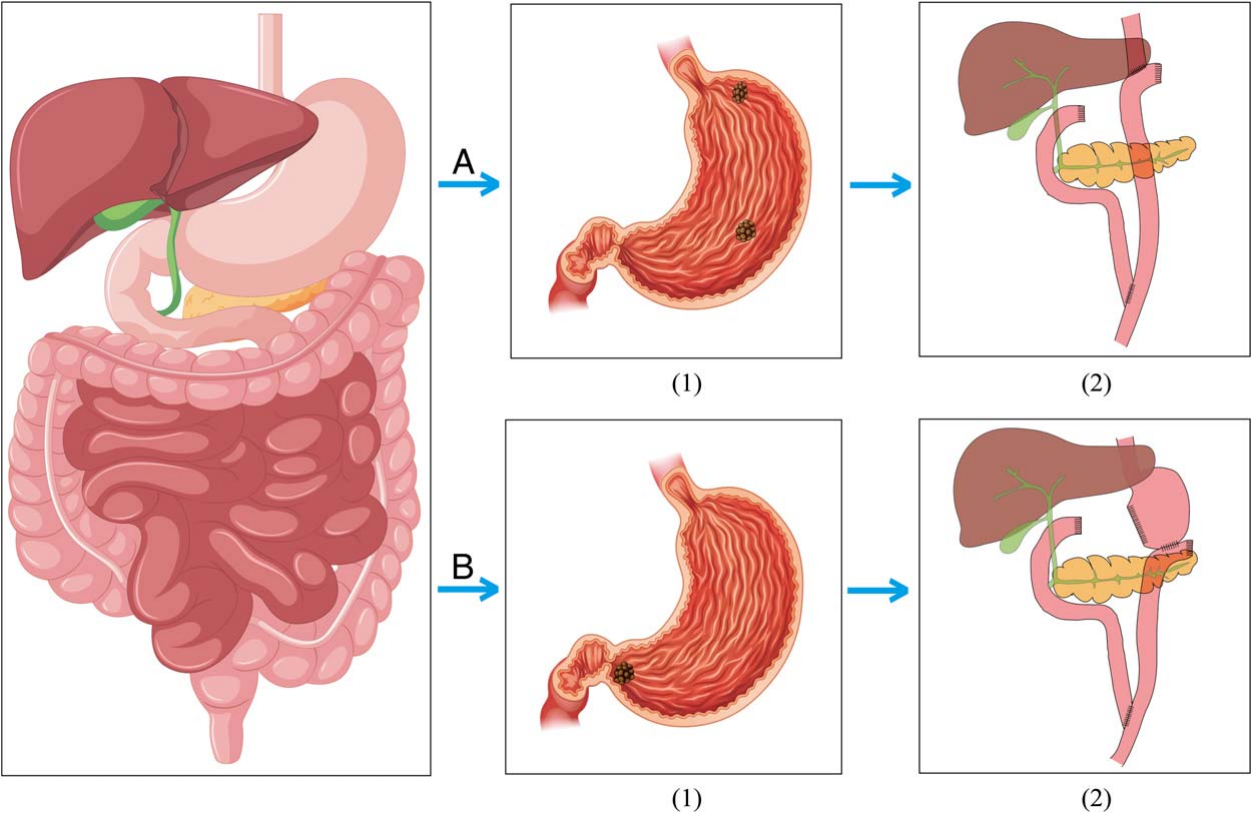
Gastrectomy procedure. A (1) Gastric fundus and body cancer; A (2) Roux-en-Y esophagojejunostomy in total gastrectomy; B (1) Distal gastric cancer; B (2) Roux-en-Y gastrojejunostomy in distal gastrectomy.

All patients underwent general anesthesia and radical gastrectomy, including resection of the primary lesion (either the entire stomach or the distal stomach), D1 or D2 standard Lymph node dissection, Roux-en-Y reconstruction, and postoperative nutritional support. In distal gastrectomy, 3/4 to 4/5 of the stomach was resected, the duodenal stump was closed, and the jejunum was severed 10–15 cm from the distal end of the Treitz ligament. The remnant stomach was anastomosed with the distal jejunum. The proximal jejunal was connected to the jejunal with located 45–60 cm below this gastrointestinal anastomosis sit. In cases of whole-stomach removal, the duodenal stump was closed, and the jejunum was severed 15–20 cm from the distal end of the Treitz ligament level, the esophagus was joined with the distal jejunum. The jejunal segment located 40 cm below this anastomosis point was linked to the proximal jejunal (Fig. 1).

### Outcome

The primary outcome was gallstone after gastrectomy, with a follow-up period through October 2023. Gallstones after gastrectomy were defined as detected following gastrectomy in patients who had no gallstones before gastrectomy. Secondary outcomes encompassed bile duct stones, acute cholecystitis, and gallbladder polyps after gastrectomy. The confirmation of all outcomes was achieved through imaging.

Patients were reviewed monthly within 6 months of surgery, semi-annually after 6 months, and annually after 2 years. Abdominal ultrasonography, regarded as the gold standard for diagnosing gallstones, exhibits strong echoes within the gallbladder and demonstrates postsounding and movement in response to changes in body position. Computed tomography (CT) or MRI, primarily used for detecting metastatic disease, could also reveal the presence of gallstones.

### Collection of covariables

Firstly, the gastrectomy range of the patients was collected, according to which the patients were divided into the distal gastrectomy group and the total gastrectomy group. Baseline data, such as sex, age, BMI, and comorbidities (hypertension, diabetes, hepatitis, fatty liver, and anemia), were systematically collected. Additionally, preoperative blood indicators, including hemoglobin (Hb), albumin (Alb), alanine aminotransferase (ALT), aspartate aminotransferase (AST), total bilirubin (TBIL), and direct bilirubin (DBIL), were assessed. Perioperative information, including mode of operation, whether hepatoduodenal ligament lymph node (No.12) dissection, whether blood transfusion, whether received neoadjuvant, postoperative pathological stage (TNM), and whether patients received at least one cycle of complete adjuvant chemotherapy were also recorded. We also collected complications after gastrectomy according to the Clavien–Dindo classification and included complications of grade II and above in our study^12,13^.

### Sample size

This study was a retrospective cohort study design, with the experimental group being the total gastrectomy group and the control group being the distal gastrectomy group, with gallstones after gastrectomy as the main evaluation index of observation. Since there is no study related to the comparison of gallstones after Roux-en-Y reconstruction after different resection ranges for gastric cancer, we calculated the sample size based on the incidence rate in one of the centers and the incidence rate of gallstones after Roux-en-Y reconstruction with total gastrectomy was about 0.39 (82/212), and that of gallstones after Roux-en-Y reconstruction with distal gastrectomy was about 0.24 (30/126). A sample of 300 subjects (150 in each group) was needed to achieve 80% efficacy. The test statistic used was a two-sided Z-test with pooled variance. The significance level of the test was 0.0500 (PASS 15.0; NCSS, LCC).

### Statistical analysis

The measured data of normal distribution show mean±SD, and the independent samples *t*-test shows to compare groups. The measured data of skew distribution indicate median (IQR), and the Wilcoxon rank-sum test shows to compare groups. The count data indicate absolute numbers and percentages, and the *χ*² test or Fisher’s exact probability method compares between groups. The Kaplan–Meier method plots the cumulative event probability curve, and the Log-Rank test assesses the difference in gallstones after gastrectomy in different groups. The COX regression proportional risk model assessed associated risk factors for gallstones following gastrectomy in gastric cancer. A balanced cohort of total and distal gastrectomies was constructed based on demographic and perioperative information using propensity score matching (PSM) (PSM ratio: 1:1, caliper: 0.2 SD of propensity scores) to reduce heterogeneity of baseline information between the two groups. The effect of gastric resection extent on gallstones after gastrectomy was investigated in different subgroups. R software (version 4.3.1, R Foundation for Statistical Computing) was used for the study, with significance differences at *P*<0.05.

## Results

### Baseline and perioperative characteristics

**Figure 2:**
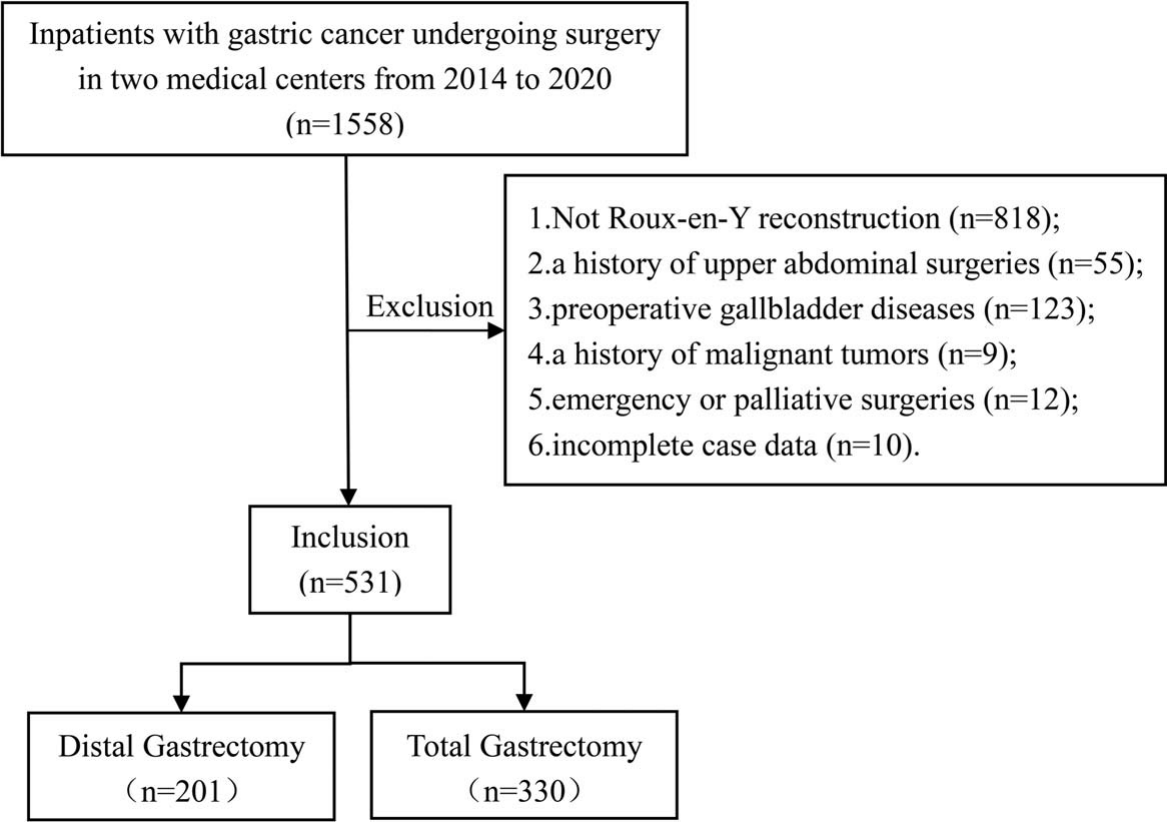
Flow chart of inclusion and exclusion.

Between January 2014 and December 2020, 1558 patients who matched the inclusion criteria for surgical treatment of gastric cancer were assessed for eligibility. After rigorous screening procedures, 1027 patients were excluded from the study (Fig. 2). The remaining patients comprised 513 and were classified into two groups based on the extent of gastrectomy: distal gastrectomy group (*n*=201) and total gastrectomy group (*n*=330). The follow-up deadline was October 2023. Table 1 displays the baseline and perioperative characteristics of the patients enrolled. Statistical differences significantly differed between the groups concerning age, surgical method, and TNM. The proportion of patients with elderly (age ≥65 years), open surgery, and III stages was higher in the total gastrectomy group than in the distal gastrectomy group. No statistical differences in other characteristics were found between the two groups.

**Table 1:** Baseline and perioperative information in the distal gastrectomy and total gastrectomy groups before propensity score matching.

		Group		
Variable	Total (*n*=531)	DG (*n*=201)	TG (*n*=330)	*P*
Age [year, *n* (%)]				0.025
<65	388 (73.07)	158 (78.61)	230 (69.70)	
≥65	143 (26.93)	43 (21.39)	100 (30.30)	
Sex, *n* (%)				0.102
Female	100 (18.83)	45 (22.39)	55 (16.67)	
Male	431 (81.17)	156 (77.61)	275 (83.33)	
BMI [kg/m^2^, *n* (%)]				0.070
<23	328 (61.77)	134 (66.67)	194 (58.79)	
≥23	203 (38.23)	67 (33.33)	136 (41.21)	
Comorbidities, *n* (%)				
Hypertension	85 (16.01)	34 (16.92)	51 (15.45)	0.656
Diabetes	35 (6.59)	14 (6.97)	21 (6.36)	0.786
Hepatitis	47 (8.85)	17 (8.46)	30 (9.09)	0.803
Fatty liver	47 (8.85)	18 (8.96)	29 (8.79)	0.947
Anemia	104 (19.59)	42 (20.90)	62 (18.79)	0.553
Preoperative blood indexes				
Hb [g/l, Mean±SD]	137.60±25.38	136.77±25.18	138.11±25.52	0.554
Alb [g/l, Mean±SD]	42.90±4.75	42.62±4.48	43.07±4.91	0.291
ALT [U/l, M (IQR)]	24.50 (16.40–40.25)	25.80 (16.60–42.90)	23.75 (16.30–38.00)	0.407
AST [U/l, M (IQR)]	23.80 (18.75–30.50)	23.30 (18.30–30.50)	23.95 (19.00–30.50)	0.543
TBIL [μmol/l, Mean±SD]	14.58±7.35	14.01±7.15	14.94±7.46	0.158
DBIL [μmol/l, Mean±SD]	5.07±2.98	4.95±3.05	5.14±2.94	0.470
Neoadjuvant, *n* (%)	72 (13.56)	22 (10.95)	50 (15.15)	0.170
Transfusion, *n* (%)	108 (20.34)	36 (17.91)	72 (21.82)	0.278
Mode of operation, *n* (%)				0.002
Open	421 (79.28)	145 (72.14)	276 (83.64)	
Laparoscopy	110 (20.72)	56 (27.86)	54 (16.36)	
Extended excision, *n* (%)	113 (21.28)	44 (21.89)	69 (20.91)	0.789
No.12 lymph node Dissection, *n* (%)	370 (69.68)	150 (74.63)	220 (66.67)	0.053
TNM, *n* (%)				0.002
I	124 (23.35)	64 (31.84)	60 (18.18)	
II	114 (21.47)	42 (20.90)	72 (21.82)	
III	280 (52.73)	89 (44.28)	191 (57.88)	
IV	13 (2.45)	6 (2.99)	7 (2.12)	
Adjuvant, *n* (%)	425 (80.04)	158 (78.61)	267 (80.91)	0.520
Length of stay, Mean±SD	19.13±5.06	19.06±5.35	19.17±4.88	0.808

Alb, albumin; ALT, alanine aminotransferase; AST, aspartate aminotransferase; DBIL, direct bilirubin; No.12 lymph node Dissection, hepatoduodenal ligament lymph node (No.12) dissection; DG, distal gastrectomy; Hb, hemoglobin; TBIL, total bilirubin; TG, total gastrectomy; TNM, postoperative pathological stage.

### Primary outcome

**Figure 3:**
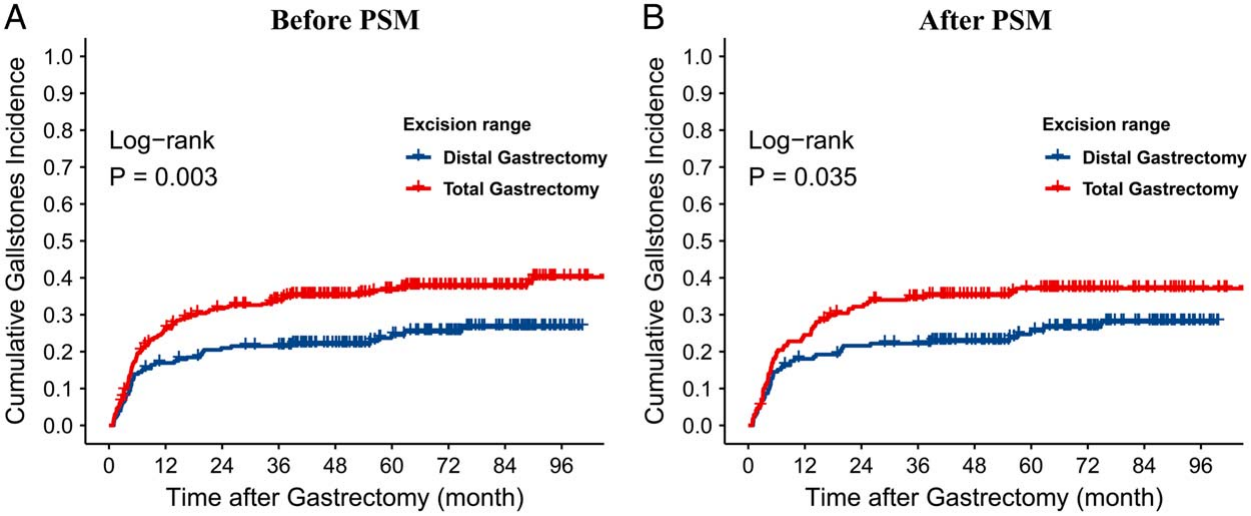
Cumulative incidence of gallstones after gastrectomy. (A) Cumulative incidence of gallstones after distal gastrectomy and total gastrectomy (P = 0.003, Log Rank test); (B) Cumulative incidence of gallstones after distal gastrectomy and total gastrectomy in PSM cohort (P = 0.035, Log Rank test).

During the study’s median follow-up period of 67.89 (64.38–71.40) months, 170 cases (32.02%) developed gallstones, with 49 cases in the distal gastrectomy group and 121 cases in the total gastrectomy (Table 2). The occurrence of gallstones in patients who underwent total gastrectomy was considerably higher than in patients who underwent distal gastrectomy within 2 years postsurgery (31.52 vs. 20.90%, *P*=0.003) (Fig. 3A).

**Table 2:** Outcomes and postoperative complications in the distal gastrectomy and total gastrectomy groups before propensity score matching.

		Group		
Variable	Total (*n*=531)	DG (*n*=201)	TG (*n*=330)	*P*
Primary outcome				
Gallstones	170 (32.02)	49 (24.38)	121 (36.67)	0.003
Secondary outcome				
Bile duct stones	4 (0.75)	2 (1.00)	2 (0.61)	1.000
Acute Cholecystitis	10 (1.88)	5 (2.49)	5 (1.52)	0.638
Gallbladder Polyp	22 (4.14)	14 (6.97)	8 (2.42)	0.011
Complications				
Overall complications (class II or higher)^a^	85 (16.01)	33 (16.42)	52 (15.76)	0.840
Serious complications (class III or higher)^a^	43 (8.1)	20 (9.95)	23 (6.97)	0.222
Incision Infection	19 (3.58)	5 (2.49)	14 (4.24)	0.291
Reflux	14 (2.64)	4 (1.99)	10 (3.03)	0.468
Stenosis	4 (0.75)	1 (0.50)	3 (0.91)	0.988
Leakage	14 (2.64)	5 (2.49)	9 (2.73)	0.867
Hemorrhage	9 (1.69)	3 (1.49)	6 (1.82)	1.000
Gastroplegia	2 (0.38)	1 (0.50)	1 (0.30)	1.000
GI obstruction	23 (4.33)	11 (5.47)	12 (3.64)	0.313
Lung Infection	8 (1.51)	3 (1.49)	5 (1.52)	1.000
Death^b^	4 (0.75)	2 (1.00)	2 (0.61)	1.000
Other	8 (1.51)	3 (1.49)	5 (1.52)	1.000

DG, distal gastrectomy; GI, gastrointestinal; TG, total gastrectomy.

a Classification according to Clavien–Dindo.

b Respiratory failure, massive hemoptysis, liver failure.

Within the first 2 years postgastrectomy, 145 gallstone cases were observed, constituting 85.29% of all gallstone cases after gastrectomy. Of these, 120 cases of gallstones were reported within the first year following gastrectomy, representing 70.59% of the overall incidence. Between 1 and 2 years after surgery, 25 cases (14.71%) were observed. Additionally, 25 cases of gallstones were reported 2 years following the gastrectomy, accounting for 14.71% of all gallstones.

### Secondary outcome

Higher incidence of gallbladder polyps in the distal gastrectomy group than in the total gastrectomy group (6.97 vs. 2.42%, *P*=0.011). There was no significant difference in bile duct stone and acute cholecystitis after gastrectomy between the distal gastrectomy group and the total gastrectomy group (Table 2). No significant differences were observed between the two groups regarding postoperative complications.

### Risk factor analysis

Univariate analyses showed that sex, BMI, the extent of gastrectomy, No.12 lymph node dissection, and adjuvant chemotherapy were correlated with the development of gallstones after Roux-en-Y reconstruction gastrectomy in gastric cancer (*P*<0.05). Multivariate analyses showed that female (HR 1.68; 95% CI: 1.18-2.40, P = 0.004), BMI ≥23 kg/m² (HR 2.00; 95% CI: 1.47–2.71, *P*<0.001), total gastrectomy (HR 1.79; 95% CI: 1.28–2.51, *P*=0.001), No.12 lymph node dissection (HR 2.36; 95% CI: 1.59–3.51, *P*<0.001), and adjuvant chemotherapy (HR 2.98; 95% CI: 1.75–5.08, *P*<0.001) were independent risk factors for gallstones after Roux-en-Y reconstruction gastrectomy in gastric cancer (Table 3).

**Table 3:** Cox regression analysis of gallstones after Roux-en-Y reconstructive gastrectomy in gastric cancer before propensity score-matched.

	Univariate		Multivariate	
Variables	HR (95% CI)	*P*	HR (95% CI)	*P*
Age (≥65 years vs. <65 years)	0.79 (0.55–1.12)	0.187		
Sex (female vs. male)	1.54 (1.09–2.19)	0.015	1.68 (1.18–2.40)	0.004
BMI (≥23 kg/m^2^ vs. <23 kg/m^2^)	1.80 (1.34–2.44)	<0.001	2.00 (1.47–2.71)	<0.001
Hypertension (yes vs. no)	0.89 (0.58–1.36)	0.583		
Diabetes (yes vs. no)	1.21 (0.68–2.12)	0.517		
Hepatitis (yes vs. no)	0.72 (0.40–1.29)	0.268		
Fatty liver (yes vs. no)	1.12 (0.68–1.85)	0.659		
Anemia (yes vs. no)	0.86 (0.57–1.28)	0.445		
Hb^a^	1.00 (1.00–1.01)	0.514		
Alb^a^	1.00 (0.97–1.03)	0.933		
ALT^a^	1.00 (0.99–1.00)	0.479		
AST^a^	0.99 (0.98–1.00)	0.279		
TBIL^a^	1.02 (1.00–1.04)	0.092		
DBIL^a^	1.02 (0.97–1.07)	0.509		
Neoadjuvant (yes vs. no)	0.98 (0.62–1.53)	0.921		
Transfusion (yes vs. no)	0.95 (0.65–1.40)	0.800		
Mode of operation (laparoscopy vs. open)	0.80 (0.54–1.19)	0.276		
Excision range (TG vs. DG)	1.64 (1.17–2.28)	0.004	1.79 (1.28–2.51)	<0.001
No.12 lymph node dissection (yes vs. no)	2.29 (1.55–3.39)	<0.001	2.36 (1.59–3.51)	<0.001
Extended excision (yes vs. no)	1.34 (0.95–1.90)	0.096		
TNM				
I	Ref			
II	1.41 (0.89–2.23)	0.142		
III	1.29 (0.87–1.91)	0.209		
IV	0.93 (0.28–3.02)	0.901		
Adjuvant (yes vs. no)	3.14 (1.85–5.34)	<0.001	2.98 (1.75–5.08)	<0.001

Alb, albumin; ALT, alanine aminotransferase; AST, aspartate aminotransferase; BMI, body mass index; DBIL, direct bilirubin; DG, distal gastrectomy; Hb, hemoglobin; TBIL, total bilirubin; TG, total gastrectomy.

No.12 lymph node Dissection, hepatoduodenal ligament lymph node (No.12) dissection; TNM, postoperative pathological stage.

a Represents preoperative blood indicators.

### Post-PSM analysis

The 1:1 PSM analysis was performed between the two groups of patients to minimize the effect of bias in demographic and perioperative information. Ultimately, 344 patients were evaluated, with each subgroup comprising 172 (Table 4). The *P* values for all variables were more significant than 0.05, indicating no statistical difference in demographic and perioperative information between the two groups of patients.

**Table 4:** Baseline and perioperative information in the distal gastrectomy and total gastrectomy groups after propensity score matching.

		Group		
Variable	Total (*n*=344)	DG (*n*=172)	TG (*n*=172)	*P*
Age [year, *n* (%)]				0.898
<65	265 (77.03)	132 (76.74)	133 (77.33)	
≥65	79 (22.97)	40 (23.26)	39 (22.67)	
Sex, *n* (%)				0.102
Female	75 (21.8)	37 (21.51)	38 (22.09)	
Male	269 (78.2)	135 (78.49)	134 (77.91)	
BMI [kg/m^2^, *n* (%)]				0.070
<23	215 (62.5)	108 (62.79)	107 (62.21)	
≥23	129 (37.5)	64 (37.21)	65 (37.79)	
Comorbidities, *n* (%)				
Hypertension	69 (20.06)	32 (18.60)	37 (21.51)	0.501
Diabetes	21 (6.1)	13 (7.56)	8 (4.65)	0.260
Hepatitis	31 (9.01)	16 (9.30)	15 (8.72)	0.851
Fatty liver	30 (8.72)	16 (9.30)	14 (8.14)	0.702
Anemia	72 (20.93)	36 (20.93)	36 (20.93)	1.000
Preoperative blood indexes				
Hb [g/l, Mean±SD]	136.39±25.29	136.52±25.39	136.26±25.27	0.925
Alb [g/l, Mean±SD]	42.75±4.80	42.77±4.43	42.72±5.15	0.932
ALT [U/l, M (IQR)]	25.50 (16.38, 41.25)	26.40 (17.50, 42.32)	22.95 (15.97, 38.30)	0.252
AST [U/l, M (IQR)]	23.45 (18.50, 30.72)	23.75 (18.50, 30.72)	23.35 (18.48, 30.72)	0.737
TBIL [μmol/l, Mean±SD]	14.34±7.06	14.19±7.11	14.49±7.03	0.692
DBIL [μmol/l, Mean±SD]	5.11±2.88	4.95±2.85	5.27±2.90	0.297
Neoadjuvant, *n* (%)	43 (12.5)	21 (12.21)	22 (12.79)	0.870
Transfusion, *n* (%)	62 (18.02)	33 (19.19)	29 (16.86)	0.575
Mode of operation, *n* (%)				0.705
Open	261 (75.87)	129 (75.00)	132 (76.74)	
Laparoscopy	83 (24.13)	43 (25.00)	40 (23.26)	
Extended excision, *n* (%)	77 (22.38)	36 (20.93)	41 (23.84)	0.518
No.12 lymph node Dissection, *n* (%)	259 (75.29)	128 (74.42)	131 (76.16)	0.708
TNM, *n* (%)				0.450
I	86 (25)	47 (27.33)	39 (22.67)	
II	79 (22.97)	35 (20.35)	44 (25.58)	
III	170 (49.42)	84 (48.84)	86 (50.00)	
IV	9 (2.62)	6 (3.49)	3 (1.74)	
Adjuvant, *n* (%)	275 (79.94)	139 (80.81)	136 (79.07)	0.686
Length of stay, Mean±SD	19.09±5.32	19.02±5.46	19.16±5.19	0.808

The prevalence of gallstones after gastrectomy was 30.81% in the PSM cohort. The incidence of gallstones after total gastrectomy was significantly higher than in the distal gastrectomy group (36.05 vs. 25.58%, *P*=0.036) (Table 5, Fig. 3B). There was no statistically significant difference between the two groups regarding postgastrectomy choledocholithiasis, acute cholecystitis, and gallbladder polyps. There was also no significant difference in postoperative complications.

**Table 5:** Outcomes and postoperative complications in the distal gastrectomy and total gastrectomy groups after propensity score matching.

		Group		
Variable	Total (n=344)	DG (n=172)	TG (n=172)	*P*
Primary outcome				
Gallstones	106 (30.81)	44 (25.58)	62 (36.05)	0.036
Secondary outcome				
Bile duct stones	2 (0.58)	2 (0.58)	2 (0.58)	0.478
Acute Cholecystitis	7 (2.03)	4 (2.33)	3 (1.74)	1.000
Gallbladder Polyp	17 (4.94)	12 (6.98)	5 (2.91)	0.082
Complications				
Overall complications (class II or higher)^a^	52 (15.12)	27 (15.70)	25 (14.53)	0.763
Serious complications (class III or higher)^a^	24 (6.98)	15 (8.72)	9 (5.23)	0.204
Incision Infection	10 (2.91)	5 (2.91)	5 (2.91)	1.000
Reflux	9 (2.62)	2 (1.16)	7 (4.07)	0.177
Stenosis	3 (0.87)	1 (0.58)	2 (1.16)	1.000
Leakage	7 (2.03)	4 (2.33)	3 (1.74)	1.000
Hemorrhage	2 (0.58)	2 (1.16)	0 (0.00)	0.478
Gastroplegia	1 (0.29)	1 (0.58)	0 (0.00)	1.000
GI obstruction	15 (4.36)	9 (5.23)	6 (3.49)	0.428
Lung Infection	8 (2.33)	3 (1.74)	5 (2.91)	0.721
Death^b^	1 (0.29)	1 (0.58)	0 (0.00)	1.000
Other	5 (1.45)	3 (1.74)	2 (1.16)	1.000

DG, distal gastrectomy; GI, gastrointestinal; TG, total gastrectomy.

a Classification according to Clavien–Dindo.

b Respiratory failure, massive hemoptysis, liver failure.

The COX regression analyses of the PSM cohort also found that female (HR 1.57; 95% CI: 1.03-2.39, P = 0.035), BMI ≥23 kg/m² (HR 1.63; 95% CI: 1.11–2.40, *P*=0.012), total gastrectomy (HR 1.51; 95% CI: 1.03–2.21, *P*=0.034), No.12 lymph node dissection (HR 2.05; 95% CI: 1.20–3.53, *P*=0.009), and adjuvant chemotherapy (HR 3.30; 95% CI: 1.63–6.71, *P*<0.001) were independent risk factors for gallstones after Roux-en-Y reconstruction gastrectomy in gastric cancer (Table 6).

**Table 6:** Cox regression analysis of gallstones after Roux-en-Y reconstructive gastrectomy in gastric cancer after propensity score-matched.

Variables	HR (95% CI)	*P*
Age (≥65 years vs. <65 years)	0.73 (0.44–1.20)	0.211
Sex (female vs. male)	1.57 (1.03–2.39)	0.035
BMI (≥23 kg/m^2^ vs. <23 kg/m^2^)	1.63 (1.11–2.40)	0.012
Hypertension (yes vs. no)	0.78 (0.49–1.25)	0.301
Diabetes (yes vs. no)	0.91 (0.42–1.97)	0.811
Hepatitis (yes vs. no)	0.45 (0.19–1.06)	0.068
Fatty liver (yes vs. no)	1.37 (0.77–2.45)	0.281
Anemia (yes vs. no)	0.77 (0.46–1.27)	0.303
Neoadjuvant (yes vs. no)	0.82 (0.44–1.52)	0.532
Transfusion (yes vs. no)	0.69 (0.39–1.19)	0.181
Mode of operation (laparoscopy vs. open)	0.80 (0.50–1.27)	0.341
Excision range (TG vs. DG)	1.51 (1.03–2.21)	0.034
No.12 lymph node dissection (yes vs. no)	2.05 (1.20–3.53)	0.009
Extended excision (yes vs. no)	1.32 (0.84–2.08)	0.224
Adjuvant (yes vs. no)	3.30 (1.63–6.71)	<0.001

**Figure 4:**
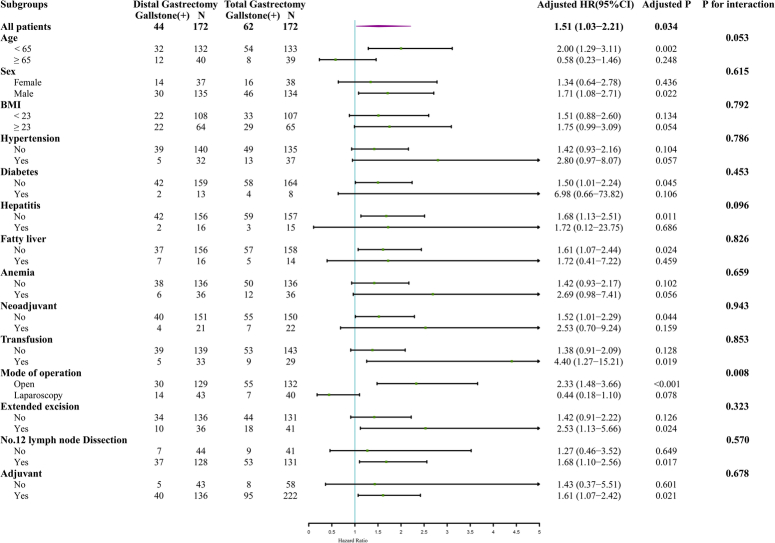
Forest plot for subgroup analysis of gallstones after gastrectomy in the PSM cohort.

Subgroup analyses of the PSM cohort showed an interaction between the surgical approach and extent of gastric resection in patients with gastric cancer on the effect of gallstones after gastrectomy (P = 0.008); Compared with distal gastrectomy, open surgery further increased the risk of gallstones after total gastrectomy. (HR 2.33; 95% CI: 1.48–3.66, *P*<0.001) (Fig. 4). The relationship between postoperative complications (such as leakage and gastrointestinal obstruction) and gallstones after gastrectomy was analyzed using data from the PSM cohort, and no statistical significance was found (S-Fig. 1, Supplemental Digital Content 2, http://links.lww.com/JS9/B816).

## Discussion

In this study, the incidence of gallstones following gastrectomy was 32.02%, significantly surpassing the prevalence observed in the general population of Asian countries^14,15^. It was noted that a significant primarily of gallstones within 2 years following surgery, with which the outcomes of prior research aligned^16^. Findings from Cox regression analysis of the post-PSM cohort indicated a higher probability of gallstone following a total gastrectomy than a distal gastrectomy in Roux-en-Y reconstructive gastrectomy for gastric cancer. A significant risk factor for gallstones after gastrectomy, as also identified in a Korean study involving 47 752 patients, was total gastrectomy^17^. During a total gastrectomy, the vagal trunk is completely severed, resulting in the loss of hepatic branch function of the vagus nerve. The hepatic branch, originating from the anterior trunk, plays a critical role in biliary function. Damage to the hepatic branch during gastrectomy can lead to impaired gallbladder contraction, the accumulation of bile salts, cholestasis, and eventually the formation of gallstones^6,18^.

A clinical investigation involving 467 early-stage gastric cancer patients confirmed that preserving the hepatic branch of the vagus nerve during laparoscopic distal gastrectomy is recommended to reduce postoperative gallstones^19^. The Japanese Gastric Cancer Treatment Guidelines 2021 (6th edition) also advocate for preserving both the hepatic and abdominal branches of the vagus nerve, as it was believed that this approach mitigates the incidence of gallstones postoperatively, facilitates early recovery and improves patients’ quality of life^20^. Total gastrectomy in Roux-en-Y reconstructive gastrectomy for gastric cancer patients in this study exhibited a higher prevalence of postoperative gallstones than distal gastrectomy. Since total gastrectomy completely severed the vagus nerve, while distal gastrectomy severed the vagus nerve to a lesser extent, the effect of the degree of vagus nerve severed on postoperative gallstones can also be confirmed laterally in this study. Thus, preserving the vagus nerve during gastrectomy in gastric cancer has a significant preventive function against postoperative gallstones.

According to a study of 1284 patients, it was found that overweight and obese individuals (BMI ≥23 kg/m^2^) had an elevated risk of gallstone formation following gastrectomy^21^. It was also denoted in our research that a high BMI exacerbates the formation of gallstones postoperatively. The primary pathophysiological mechanism that drives gallstone formation is the hypokinesis of the gallbladder, a condition that inhibits gallbladder emptying, causing cholestasis and bile salt deposition, thereby paving the way for gallstone development^22^. This mechanism can be observed in obese individuals with significant weight loss in men^23^. The association between high BMI and postoperative gallstone formation could be due to the increased visceral and omental fat content in overweight and obese patients, inducing more significant postoperative weight loss. Besides, obesity is associated with the formation of gallstones, as it enhances cholesterol synthesis and excretion in the liver, leading to cholesterol supersaturation in the gallbladder, and the formation of cholesterol stones^24^.

We also observed a significant increase in gallstone incidence among female patients postgastrectomy. Possible explanations include females’ inherent predisposition to gallstone formation, or that our study focused on a population undergoing Roux-en-Y reconstruction, or it may be due to the small proportion of women in our included population^24^. Therefore, there is a need to further investigate the relationship between gender and gallstones after gastrectomy.

A retrospective study of 805 patients found a direct correlation between the extent of lymph node dissection and the incidence of gallstones following gastrectomy^25^. Patients who underwent D2 or D2+ lymph node dissection had an increased risk of developing symptomatic gallstones postgastrectomy, potentially requiring revisional cholecystectomy. However, due to variations in D2 lymph node dissection scope between total and distal gastrectomy, our study only examined the influence of hepatoduodenal ligament lymph node (No.12 lymph node) on gallstone formation postoperatively. The results indicated that No.12 lymph node dissection was an independent risk factor for developing gallstones after gastrectomy. The potential mechanism may involve edema, inflammation, and adhesions surrounding the bile duct after hepatoduodenal ligament lymph node dissection, which leading to the obstruction of bile outflow and the concentration of bile in the gallbladder, which may promote gallstone formation^26^.

A study of 561 patients found that those not receiving adjuvant chemotherapy had a higher prevalence of postoperative gallstones^27^. On the contrary, our research indicated that adjuvant chemotherapy might increase gallstone risk after gastrectomy. Postoperative adjuvant chemotherapy is a valuable approach to lowering the risk of tumor recurrence and enhancing survival rates among patients with high-risk tumors following surgical intervention. Adjuvant chemotherapy could be related to changes in intestinal flora, which plays a crucial role in gallstone formation^28–31^. Due to the unclear relationship between adjuvant chemotherapy and gallstones after gastrectomy for gastric cancer, further research is needed.

In the post-PSM cohort, subgroup analysis revealed that open surgery increased further the risk of gallstones after total gastrectomy. This may be related to the complete severing of the vagus nerve in open total gastrectomy as well as postoperative adhesions in the surrounding tissues, which in turn have a greater impact on the contractile function of the gallbladder, among other things. Diabetes is a potential risk factor for gallstone development following gastrectomy with gastric cancer in two cohort studies^32,33^. However, our study did not find a correlation between preoperative comorbidities (including hypertension, diabetes, and others) and the formation of gallstones after gastrectomy. Further research is necessary to understand the relationship between preoperative comorbidities and gallstone development after gastrectomy. We categorized postgastrectomy complications according to Clavien–Dindo and examined potential associations between leakage, gastrointestinal obstruction, and gallstones after gastrectomy in a post-PSM cohort using a log-rank test. The results showed no association, which may be due to the limited sample size. More extensive studies with larger sample sizes are needed to determine the association.

Prevention of gallstones after gastrectomy consists mainly of postoperative prophylactic use of ursodeoxycholic acid and prophylactic cholecystectomy. Prophylactic use of ursodeoxycholic acid after gastrectomy reduces the incidence of gallstones after gastrectomy and the likelihood of cholecystectomy^34,35^. In a study recommended that cholecystectomy be performed concurrently with gastrectomy in elderly patients with Roux-en-Y reconstruction^36^. Additionally, a study of 2383 patients undergoing laparoscopic sleeve gastrectomy for weight loss found that concurrent cholecystectomy was safe and necessary for symptomatic gallstones, but prophylactic cholecystectomy was deemed unnecessary in the absence of gallstones^37^. It has also been suggested that individualized prophylactic cholecystectomy for gallstones after gastric cancer surgery will be the focus of future research^38^. Prophylactic cholecystectomy during gastrectomy for gastric cancer remains controversial.

It is worth noting that Prophylactic cholecystectomy is performed to avoid repeat cholecystectomy after gastrectomy due to symptomatic gallstones. However, in the post-PSM cohort of this study, the incidence of gallstones after gastrectomy was 30.81%, while symptomatic gallstones (acute cholecystitis) accounted for only 6.6% of gallstones (7/106). Therefore, preservation of the gallbladder, along with avoidance of vagal nerve injury during gastrectomy and postoperative prophylactic use of ursodeoxycholic acid, may be a more favorable option to reduce the development of symptomatic gallstones in patients with high-risk factors.

There are limitations in the present study. First, some patients underwent only CT during the follow-up period, which could have led to negative stones undetected and affected the overall incidence of stones. Second, this study is retrospective, and bias may exist. In a subsequent multicenter prospective study, a larger patient cohort will be utilized to assess the incidence and associated risk factors of gallstones after Roux-en-Y reconstructive gastrectomy in gastric cancer, as well as further investigate preventive measures.

## Conclusion

Gallstones occurred in 32.02% of patients after Roux-en-Y reconstruction gastrectomy in gastric cancer and primarily within 2 years after gastrectomy. Females, BMI ≥23 kg/m^2^, total gastrectomy, No.12 lymph node dissection, and adjuvant chemotherapy were risk factors for gallstones after Roux-en-Y reconstructive gastrectomy. Patients with the above risk factors should close follow-up after surgery to avoid the development of symptomatic gallstones.

## Ethical approval

The ethics committee of The First Hospital of Lanzhou University (LDYYLL2023-377) approved the study.

## Consent

This study is retrospective and does not involve patient images and other private information, which has been approved by the ethics committee of the hospital.

## Sources of funding

This study was supported by the National Natural Science Foundation of China (32160255); the Natural Science Foundation of Gansu Province: (22JR5RA898). Medical Innovation and Development Project of Lanzhou University (lzuyxcx-2022-157).

## Author contribution

B.Z.: data curation, methodology, software, writing – original draft, and writing – review and editing; P.N. and Y.L.: data curation, methodology, and writing – original draft; Z.M. and G.M.: data curation and methodology; Y.W. and J.Z.: data curation and supervision; Y.M. and J.Z.: data curation and methodology; P.Y.: data curation, methodology, and supervision; N.J., X.Z., and L.T.: data curation; L.L.: conceptualization, methodology, and supervision; J.Y.: conceptualization, supervision, methodology, and writing – review and editing; W.M.: conceptualization, funding acquisition, investigation, methodology, project administration, supervision, and writing – review and editing. B.Z., P.N., and Y.L.: contributed to this work equally.

## Conflicts of interests disclosure

The authors declared that no competing interests exist.

## Research registration unique identifying number (UIN)

This study was registered at clinicaltrials (NCT05965466). (https://clinicaltrials.gov/ct2/show/NCT05965466?term=NCT05965466&draw=2&rank=1).

## Guarantor

Wenbo Meng, MD, PhD, The First Hospital of Lanzhou University, Lanzhou, Gansu 730030, People’s Republic of China. E-mail: mengwb@lzu.edu.cn.

## Data availability statement

The data that support the study findings are available upon reasonable request from the corresponding authors [Linzhi Lu, Jinqiu Yuan, and Wenbo Meng].

## Provenance and peer review

Not commissioned, externally peer-reviewed.

## Supplementary Material

**Figure:**
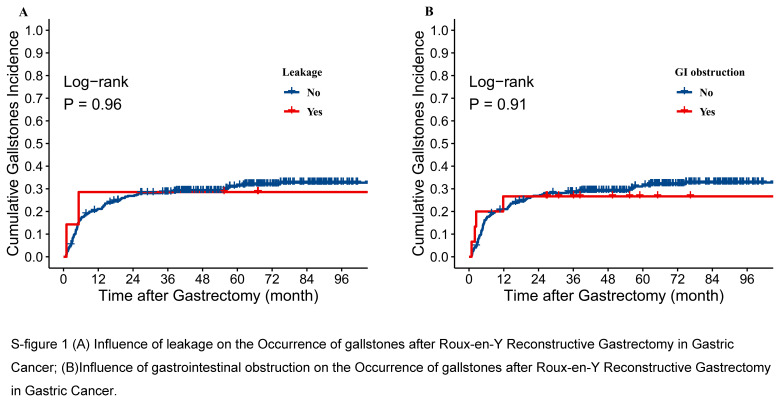


## References

[1] Sung H Ferlay J Siegel RL. Global Cancer Statistics 2020: GLOBOCAN estimates of incidence and mortality worldwide for 36 cancers in 185 countries. CA Cancer J Clin 2021;71:209–249. 33538338 10.3322/caac.21660

[2] Hikage M Hato S Uemura K. Late complication after gastrectomy for clinical stage I cancer: supplementary analysis of JCOG0912. Surg Endosc 2023;37:2958–2968. 36512122 10.1007/s00464-022-09804-8

[3] Hauters P de Neve de Roden A. Cholelithiasis: a serious complication after total gastrectomy. Br J Surg 1988;75:899–900. 3179669 10.1002/bjs.1800750923

[4] Liang TJ Liu SI Chen YC. Analysis of gallstone disease after gastric cancer surgery. Gastric Cancer 2017;20:895–903. 28154944 10.1007/s10120-017-0698-5

[5] Fujita S Kimata M Matsumoto K. Important risk factors for gallstones after laparoscopic gastrectomy: a retrospective study. BMC Surg 2022;22:5. 34996411 10.1186/s12893-021-01458-y PMC8742379

[6] Ihasz M Griffith CA. Gallstones after vagotomy. Am J Surg 1981;141:48–50. 7457726 10.1016/0002-9610(81)90010-6

[7] Rieu PN Jansen JB Hopman WP. Effect of partial gastrectomy with Billroth II or Roux-en-Y anastomosis on postprandial and cholecystokinin-stimulated gallbladder contraction and secretion of cholecystokinin and pancreatic polypeptide. Dig Dis Sci 1990;35:1066–1072. 2390921 10.1007/BF01537576

[8] Smyth EC Nilsson M Grabsch HI. Gastric cancer. Lancet 2020;396:635–648. 32861308 10.1016/S0140-6736(20)31288-5

[9] Asaka M Kato M Sakamoto N. Roadmap to eliminate gastric cancer with Helicobacter pylori eradication and consecutive surveillance in Japan. J Gastroenterol 2014;49:1–8. 24162382 10.1007/s00535-013-0897-8 PMC3895201

[10] Nishizaki D Ganeko R Hoshino N. Roux-en-Y versus Billroth-I reconstruction after distal gastrectomy for gastric cancer. The Cochrane Database Syst Rev 2021;9:Cd012998. 34523717 10.1002/14651858.CD012998.pub2 PMC8441595

[11] Mathew G Agha R Albrecht J. STROCSS 2021: strengthening the reporting of cohort, cross-sectional and case-control studies in surgery. Int J Surg 2021;96:106165. 34774726 10.1016/j.ijsu.2021.106165

[12] Clavien PA Sanabria JR Strasberg SM. Proposed classification of complications of surgery with examples of utility in cholecystectomy. Surgery 1992;111:518–526. 1598671

[13] Dindo D Demartines N Clavien P-A. Classification of surgical complications: a new proposal with evaluation in a cohort of 6336 patients and results of a survey. Ann Surg 2004;240:205–213. 15273542 10.1097/01.sla.0000133083.54934.ae PMC1360123

[14] Colvin HS Kimura T Iso H. Risk factors for gallstones and cholecystectomy: a large-scale population-based prospective cohort study in Japan. Dig Dis 2022;40:385–393. 34023821 10.1159/000517270

[15] Song Y Ma Y Xie FC. Age, gender, geographic and clinical differences for gallstones in China: a nationwide study. Ann Transl Med 2022;10:735. 35957733 10.21037/atm-21-6186 PMC9358507

[16] Fukagawa T Katai H Saka M. Gallstone formation after gastric cancer surgery. J Gastrointestinal Surg 2009;13:886–889. 10.1007/s11605-009-0832-8 19219514

[17] Seo GH Lim CS Chai YJ. Incidence of gallstones after gastric resection for gastric cancer: a nationwide claims-based study. Ann Surg Treat Res 2018;95:87–93. 30079325 10.4174/astr.2018.95.2.87 PMC6073047

[18] Patankar R Ozmen MM Bailey IS. Gallbladder motility, gallstones, and the surgeon. Dig Dis Sci 1995;40:2323–2335. 7587810 10.1007/BF02063233

[19] Wang CJ Kong SH Park JH. Preservation of hepatic branch of the vagus nerve reduces the risk of gallstone formation after gastrectomy. Gastric Cancer 2021;24:232–244. 32705445 10.1007/s10120-020-01106-z

[20] Association JGC. Japanese Gastric Cancer Treatment Guidelines 2021 (6th edition). Gastric Cancer 2023;26:1–25. 36342574 10.1007/s10120-022-01331-8 PMC9813208

[21] Park DJ Kim KH Park YS. Risk factors for gallstone formation after surgery for gastric cancer. J Gastric Cancer 2016;16:98–104. 27433395 10.5230/jgc.2016.16.2.98 PMC4944009

[22] Reshetnyak VI. Concept of the pathogenesis and treatment of cholelithiasis. World J Hepatol 2012;4:18–34. 22400083 10.4254/wjh.v4.i2.18 PMC3295849

[23] Tsai CJ Leitzmann MF Willett WC. Weight cycling and risk of gallstone disease in men. Arch Intern Med 2006;166:2369–2374. 17130391 10.1001/archinte.166.21.2369

[24] Lammert F Gurusamy K Ko CW. Gallstones. Nat Rev Dis Primers 2016;2:16024. 27121416 10.1038/nrdp.2016.24

[25] Akatsu T Yoshida M Kubota T. Gallstone disease after extended (D2) lymph node dissection for gastric cancer. World J Surg 2005;29:182–186. 15654665 10.1007/s00268-004-7482-5

[26] Kobayashi T Hisanaga M Kanehiro H. Analysis of risk factors for the development of gallstones after gastrectomy. Br J Surg 2005;92:1399–1403. 16078296 10.1002/bjs.5117

[27] Lee YW Kim A Han M. Risk factors for gallbladder stone formation after gastric cancer surgery. J Gastric Cancer 2019;19:417–426. 31897344 10.5230/jgc.2019.19.e37 PMC6928081

[28] Erawijantari PP Mizutani S Shiroma H. Influence of gastrectomy for gastric cancer treatment on faecal microbiome and metabolome profiles. Gut 2020;69:1404–1415. 31953253 10.1136/gutjnl-2019-319188 PMC7398469

[29] Shuwen H Xi Y Yuefen P. Effects of postoperative adjuvant chemotherapy and palliative chemotherapy on the gut microbiome in colorectal cancer. Microb Pathog 2020;149:104343. 32562813 10.1016/j.micpath.2020.104343

[30] Hu H Shao W Liu Q. Gut microbiota promotes cholesterol gallstone formation by modulating bile acid composition and biliary cholesterol secretion. Nat Commun 2022;13:252. 35017486 10.1038/s41467-021-27758-8 PMC8752841

[31] Dan WY Yang YS Peng LH. Gastrointestinal microbiome and cholelithiasis: current status and perspectives. World J Gastroenterol 2023;29:1589–1601. 36970590 10.3748/wjg.v29.i10.1589 PMC10037248

[32] Jun KH Kim JH Kim JJ. Retrospective analysis on the gallstone disease after gastrectomy for gastric cancer. Gastroenterol Res Pract 2015;2015:827864. 26180526 10.1155/2015/827864 PMC4477116

[33] Paik KH Lee JC Kim HW. Risk factors for gallstone formation in resected gastric cancer patients. Medicine 2016;95:e3157. 27082555 10.1097/MD.0000000000003157 PMC4839799

[34] Lee SH Jang DK Yoo MW. Efficacy and safety of ursodeoxycholic acid for the prevention of gallstone formation after gastrectomy in patients with gastric cancer: the PEGASUS-D randomized clinical trial. JAMA Surg 2020;155:703–711. 32584935 10.1001/jamasurg.2020.1501 PMC7301302

[35] Mulliri A Menahem B Alves A. Ursodeoxycholic acid for the prevention of gallstones and subsequent cholecystectomy after bariatric surgery: a meta-analysis of randomized controlled trials. J Gastroenterol 2022 Aug;57:529–539. 35704084 10.1007/s00535-022-01886-4

[36] Wu CH Huang KH Chen MH. Comparison of the long-term outcome between Billroth-I and Roux-en-Y reconstruction following distal gastrectomy for gastric cancer. J Gastrointest Surg 2021;25:1955–1961. 33205309 10.1007/s11605-020-04867-1

[37] Raziel A Sakran N Szold A. Concomitant cholecystectomy during laparoscopic sleeve gastrectomy. Surg Endosc 2015;29:2789–2793. 25480625 10.1007/s00464-014-4010-z

[38] Liu H Liu J Xu W. Prophylactic cholecystectomy: a valuable treatment strategy for cholecystolithiasis after gastric cancer surgery. Front Oncol 2022;12:897853. 36176409 10.3389/fonc.2022.897853 PMC9513465

